# Nanoscopic substructures of raft-mimetic liquid-ordered membrane domains revealed by high-speed single-particle tracking

**DOI:** 10.1038/srep20542

**Published:** 2016-02-10

**Authors:** Hsiao-Mei Wu, Ying-Hsiu Lin, Tzu-Chi Yen, Chia-Lung Hsieh

**Affiliations:** 1 Institute of Atomic and Molecular Sciences, Academia Sinica, Taipei 10617, Taiwan

## Abstract

Lipid rafts are membrane nanodomains that facilitate important cell functions. Despite recent advances in identifying the biological significance of rafts, nature and regulation mechanism of rafts are largely unknown due to the difficulty of resolving dynamic molecular interaction of rafts at the nanoscale. Here, we investigate organization and single-molecule dynamics of rafts by monitoring lateral diffusion of single molecules in raft-containing reconstituted membranes supported on mica substrates. Using high-speed interferometric scattering (iSCAT) optical microscopy and small gold nanoparticles as labels, motion of single lipids is recorded via single-particle tracking (SPT) with nanometer spatial precision and microsecond temporal resolution. Processes of single molecules partitioning into and escaping from the raft-mimetic liquid-ordered (L_o_) domains are directly visualized in a continuous manner with unprecedented clarity. Importantly, we observe subdiffusion of saturated lipids in the L_o_ domain in microsecond timescale, indicating the nanoscopic heterogeneous molecular arrangement of the L_o_ domain. Further analysis of the diffusion trajectory shows the presence of nano-subdomains of the L_o_ phase, as small as 10 nm, which transiently trap the lipids. Our results provide the first experimental evidence of non-uniform molecular organization of the L_o_ phase, giving a new view of how rafts recruit and confine molecules in cell membranes.

Phase transition and domain formation in biological membranes are fascinating phenomena that play critical roles in many membrane functions^1^,^2^,^3^,^4^. Among the various phase separations, coexistence of liquid-ordered (L_o_) and liquid-disordered (L_d_) phases is of particular interest as it underlies the concepts of lipid rafts in biological membranes^5^,^6^. Lipid rafts are nanoscopic membrane domains enriched in saturated lipids of high melting temperature (e.g., sphingolipids) and cholesterol, which actively participate in various biological processes, including signaling transduction^5^, membrane trafficking^6^ and viral entry^7^. Previous studies have suggested that the relatively ordered molecular structure of lipid rafts can selectively recruit and trap lipids and membrane proteins of similar molecular properties^8^,^9^, and thus, rafts are postulated as local reaction centers for protein clustering and activations^5^,^6^. Characterization of lipid raft domains is therefore of great importance in order to understand fundamental regulation mechanism of membrane mobility and its relationship to membrane functions.

Since the molecular composition of cell membranes is highly diverse, inherent properties of lipid rafts have been extensively studied in simplified model membranes of ternary lipid compositions where the mimetic rafts of L_o_ phase coexists with non-raft L_d_ phase^10^,^11^,^12^,^13^. In this simple system, it has been found that the L_o_ phase consists largely of saturated lipids of high melting temperature and cholesterol, sharing many properties of lipid rafts in the cell membranes^10^. The term “liquid-ordered” is coined to describe a special phase of lipids with ordered and extended acyl chains, but it still exhibits the liquid-like lateral and rotational mobility^11^. Cholesterol plays a unique role in the formation of L_o_ phase by disturbing the translational order in the crystalline (gel) state of the lipids^14^. Despite many properties of the L_o_ phase have been learned^15^,^16^, their implications to rafts in cell membranes remain unclear. Previous measurements indicated that the putative rafts in cell plasma membranes are nano-sized^5^,^6^. Therefore, ideally, characterization of the L_o_ phase should be performed with nanometer spatial resolution so that it provides nanoscopic details that are more relevant to rafts in the cell membranes. Unfortunately, such nanoscopic measurement in aqueous environment has been challenging. Furthermore, biological membrane is a dynamic fluidic system in which molecules undergo fast lateral diffusion due to thermal fluctuations: single molecules diffuse by a few nanometers in one microsecond in the membrane. Therefore, interaction between individual membrane molecules with the L_o_ domains at the nanoscale is expected to be very transient and its detection requires high temporal resolution of microseconds. Finally, single-molecule sensitivity is another desired capability in order to observe short-lived and rare interactions that can be obscured via ensemble average. Putting together, investigation of molecular dynamics of rafts calls for a technique with single-molecule sensitivity, nanometer spatial resolution and microsecond temporal resolution, which has been technically challenging for existing techniques.

Scattering-based single-particle tracking (SPT) is a promising technique for high-speed localization of single membrane molecules. Using 40 nm gold nanoparticles (GNPs) as labels with bright-field detection, single lipids and membrane proteins were tracked in the plasma membrane with 17 nm spatial precision at 40 kHz from which membrane compartmentalization of hundreds of nanometers was discovered^17^. Recently, a novel concept of interferometric detection of scattering signal (iSCAT) has been introduced that shows promise of further improving the sensitivity and spatiotemporal resolution of scattering-based SPT^18^,^19^,^20^,^21^,^22^,^23^,^24^,^25^. The interferometric detection makes it possible to circumvent the limitation of signal-to-noise ratio (SNR) set by the electronic noise of high-speed detectors, reaching the optimal sensitivity required for super localization of very small particle via intrinsic scattering^24^. By iSCAT microscopy, detection and localization of single nanoparticles, ranging from metallic particles to virus particles have been demonstrated^20^,^21^,^22^,^23^,^24^,^25^. The stable and unsaturated scattering signal allows for long-term detection with unlimited observation time. In this work, we use ultrahigh-speed iSCAT SPT at 50 kHz to investigate single-molecule diffusion in raft-containing reconstituted bilayer membranes. The capability of tracking single lipid molecules at the nanoscale via 20 nm GNP tags without introducing labeling artifacts is carefully examined. The iSCAT microscopy allows for continuous observation of single lipid molecules partitioning into and escaping from the raft-mimetic L_o_ domains with nanometer spatial precision and microsecond temporal resolution. From the detailed trajectories of single lipids exploring the raft-containing membranes, we observe deviation from Brownian motion of lipid molecules in the L_o_ domain in the microsecond timescale, showing the first experimental evidence of the nanoscopic heterogeneous molecular arrangement of the L_o_ phase. Our finding also reveals the existence of nano-subdomains in the L_o_ phase that strongly influence motion of molecules. These nano-subdomains are expected to be critical for recruiting and confining raft-associated molecules in lipid rafts of cell membranes.

## Results

### High-speed nanoscopic tracking of single lipid molecules by iSCAT microscopy

**Figure 1 f1:**
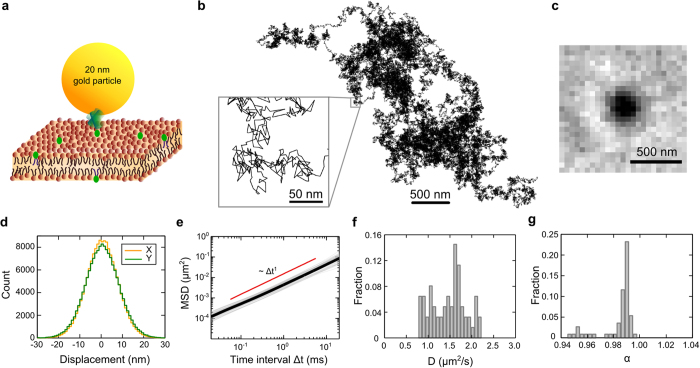
Visualization of single lipid diffusion in homogeneous bilayer membrane via iSCAT SPT with 3 nm spatial precision at 50 kHz. (**a**) Schematics of 20 nm GNP attaching to single lipid molecule at the headgroup in a membrane for high-speed iSCAT tracking. The particle and the bilayer membrane are plotted at the same scale. (**b**) Diffusion trajectory of single lipid molecule in a homogeneous membrane recorded at 50 kHz for 3.1 s (see also Movie S1). (**c**) An iSCAT snapshot of a 20 nm GNP diffusing on the membrane captured at 50 kHz. The spot size is ~300 nm (full width at half maximum), and the SNR is 20. The center of the spot is determined by SPT and the precision of determining the center position is 3 nm. (**d**) Step size distributions in two lateral directions at 20 μs timescale both show perfect Gaussian distributions. (**e**) Time-average MSD of 62 diffusion trajectories (grey lines) and their ensemble average (thick black line), showing uniform Brownian diffusion. The red line indicates the linear dependence of the MSD on the time interval Δt as for perfect Brownian diffusion. (**f**) Histogram of the measured diffusion rate (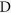 = 1.48 ± 0.39 μm^2^ s^−1^). (**g**) Histogram of measured anomalous exponent α. Narrow peak around α = 1 indicates the diffusion in homogeneous membrane is nearly Brownian.

Small GNP of 20 nm in diameter is used to label lipid molecule in the membrane via streptavidin and biotin for optical imaging and tracking (Fig. 1a). The iSCAT microscope (see Methods and Fig. S1) captures scattering image of single GNP via interferometry at 50 kHz. Benchmark measurements were performed by tracking single probe lipid (biotin-cap-DPPE) diffusing laterally in a homogeneous DiPhyPC membrane of L_d_ phase prepared on an atomically flat mica substrate (see Methods). We chose DiPhyPC instead of other commonly used unsaturated phospholipids in order to avoid photo-oxidation effect of the lipid molecules that may modify the mobility of the lipids^26^. Multiple particles can be simultaneously visualized on the membrane (see Movie S1), and their diffusion trajectories were obtained by SPT (see Methods). Figure 1b shows a representative diffusion trajectory consisting of more than 155,000 steps. The iSCAT image of 20 nm GNP has a SNR ~20 (Fig. 1c), providing a spatial localization precision of 3 nm (Fig. S2). The long and accurate trajectory together with 20 μs temporal resolution allows us to analyze its diffusion characteristics with high accuracy. We observe perfect Gaussian step size distributions in two lateral directions, implying the measurement provides sufficient data for statistical analysis (Fig. 1d). To assure the labeling scheme and the measurement are reliable, we collected a large amount of diffusion data of 62 trajectories (each of which is longer than 6,000 steps) and calculated their individual time-averaged mean squared displacements (MSD) as a function of time interval Δt and their diffusion rates 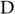 (see Fig. 1e[Fig f1]f and Methods). We measured similar diffusion rates from individual particles (1.48 ± 0.39 μm^2^ s^−1^), suggesting high uniformity of the membrane and the particles. To analyze the diffusion characteristics, the MSD was fitted by a model of anomalous diffusion^27^:


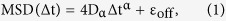


where 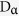 is the anomalous transport coefficient, 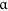 is the anomalous exponent, and 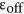 is an offset due to the dynamic localization error^28^. The histogram of the fitted values of 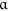 shows a narrow peak at 0.99 (Fig. 1g), implying the diffusion was nearly Brownian (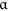 = 1 when diffusion is perfectly Brownian). The observed Brownian diffusion indicates that the membrane is homogeneous over the length scales from nanometers to micrometers and the supporting substrate does not introduce spatially heterogeneous effects to the membrane. Brownian diffusion of biotin-cap-DPPE was also observed in homogeneous membranes of unsaturated phospholipids DOPC via GNP labeling and iSCAT SPT (data not shown). We further verified that the mass loading of 20 nm GNP was negligible, as the GNP showed the same diffusion rate as single dye-labeled streptavidin when labeled to biotin-cap-phospholipid (Fig. S3). More about the validity of tracking single lipid molecules by localizing GNP labels are discussed in Supplementary Information.

### Diffusion of single lipid molecules in raft-containing membranes

**Figure 2:**
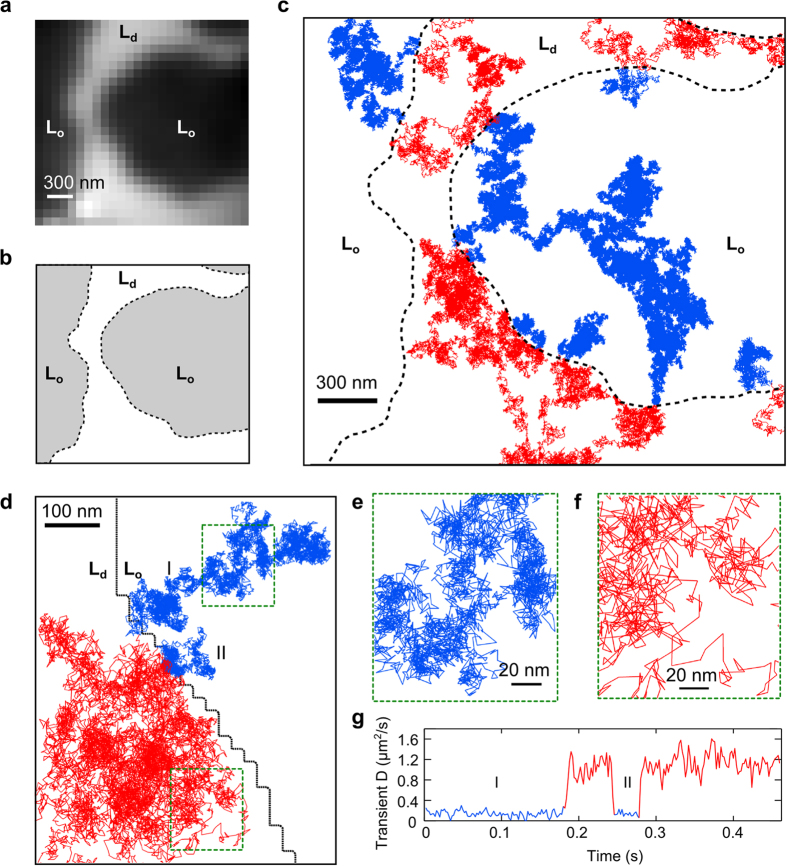
Continuous observation of single lipid molecule diffusion in raft-containing membrane at 50 kHz. (**a**) Fluorescence raw image of micron-sized raft-mimetic L_o_ domains (appear as dark areas) in the membrane. (**b**) Areas of the L_o_ and L_d_ domains defined from the fluorescence image. (**c**) Ten representative diffusion trajectories of single lipid molecules in the raft-containing membrane recorded at 50 kHz where each of them explores both the L_d_ and L_o_ domains. The parts of trajectories in the L_d_ and L_o_ domains are colored in red and blue respectively, while the domain boundaries are marked by the dashed lines. (**d**) A segment of diffusion trajectory of a lipid molecule repeatedly leaving from and entering the raft domain (see Movie S2). Domain boundary is plotted as the black line. The lipid was initially in the L_o_ domain (marked as I); it left the L_o_ domain and re-entered the L_o_ domain shortly (marked as II), and it left the L_o_ domain again at the end. (**e,f**) Close-up views of the trajectory shown in (**d**), in the L_o_ domain (**e**) and in the L_d_ domain (**f**) respectively, showing distinct diffusion behaviors. (**g**) Transient diffusion rate of the lipid calculated from the trajectory in (**d**). The diffusion rates in the L_o_ and L_d_ domains are very different: slow diffusion of 0.13 μm^2^ s^−1^ and fast diffusion of 1.19 μm^2^ s^−1^ were measured in the L_o_ and L_d_ domains respectively.

Using high-speed iSCAT SPT, we investigated diffusion of single lipid molecules in raft-containing membranes at high spatiotemporal resolution. The raft-containing bilayer membranes were prepared from a lipid mixture of DiPhyPC/DPPC/cholesterol (40:40:20 mol%) and a trace amount of lipophilic dye DiI-C18 (see Methods). Micro-sized L_o_ domains were observed via fluorescence (Fig. 2a) from which the domain boundaries were determined (Fig. 2b). We noticed that the L_o_ domains can slowly drift, rearrange, and merge over time as reported previously^29^. Within our short observation time of one minute, no changes of the shape and location of the L_o_ domains were observed in the fluorescence image. Interestingly, the L_o_ domains also appear in iSCAT image with low contrast (~1%) due to the slight mismatch of refractive index of the L_d_ and L_o_ phases, as reported recently^30^. We then added GNPs to label the probe lipid (biotin-cap-DPPE) and followed their motions via iSCAT SPT at 50 kHz. Each high-speed iSCAT recording took a few seconds, and we were able to record many videos within one minute before the shape of the L_o_ domain changed noticeably. Note that the precise localization of the GNP was performed on background-free iSCAT images where the non-uniform stationary background due to the illumination, the membrane domains and the heterogeneous pixel responses was removed (Methods). The recorded diffusion trajectories of the single probe lipids were superimposed on the fluorescence image of the same sample area indicating the location of the raft-mimetic L_o_ domains (Fig. 2c). The stable scattering signal from the GNP provides the opportunity to capture precise and continuous diffusion trajectories of single probe lipids exploring both the L_d_ and L_o_ domains, and therefore enables investigation of how the L_o_ domain influences diffusion of lipid molecules. In our recorded data, more than 90% of the trajectories explore both the L_d_ and L_o_ domains. We emphasize that the capability of observing single particle exploring both domains is valuable as it allows for direct comparison of diffusion characteristics of the same particle in both domains, and therefore possible systematic artifacts introduced by the inevitable differences between individual probe nanoparticles (e.g., size, shape, orientation of the crosslinker molecule, etc.) can be avoided. Figure 2d shows a segment of the diffusion trajectory of a lipid molecule repeatedly escaping from and entering the L_o_ domain (see also Movie S2). Close-up views of the trajectories in Fig. 2e,f reveal distinct diffusion behaviors in the L_o_ and L_d_ domains. It is apparent that the transient diffusion rate of the probe lipid changes abruptly when it crosses the domain boundary (Fig. 2g, see Methods for the calculation of transient diffusion rate). From this trajectory, we observe fast diffusion of 1.19 μm^2^ s^−1^ in the L_d_ domain and slow diffusion of 0.13 μm^2^ s^−1^ in the L_o_ domain. Our experiment also clearly illustrates the fact that while the morphology of the L_o_ domain does not change within the observation time, individual molecules in the membrane undergo rapid diffusive motion, and they even constantly cross the domain boundary.

### Anomalous molecular diffusion in the L_o_ phase

**Figure 3:**
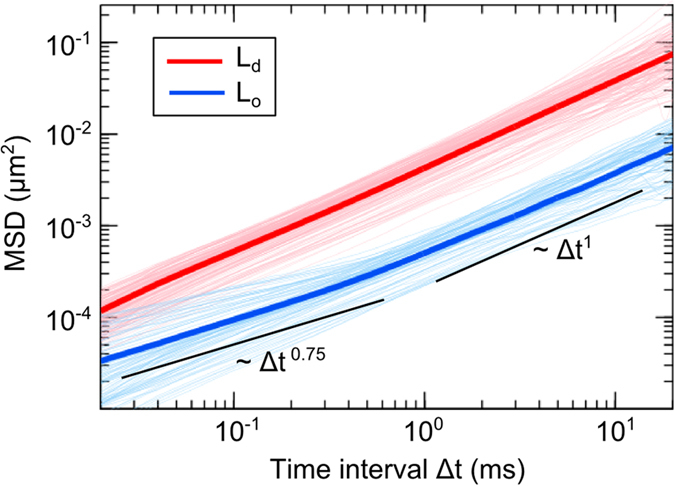
Time-averaged MSD measured in the L_d_ and L_o_ domains from 20 μs to 20 ms. 166 and 88 trajectories were measured from the L_d_ and L_o_ domains respectively, and their MSDs were individually calculated and plotted. The red and blue thick lines are their corresponding ensemble averages. In this double logarithmic plot of MSD, the slope represents the value of anomalous exponent 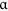. Two black solid lines show the slopes of 0.75 and one. Single lipid molecules diffuse freely in the L_d_ domain, as the slopes are nearly one. On the other hand, the lipid molecules undergo anomalous subdiffusion in the L_o_ domains at the microsecond timescale, indicating the molecular diffusion in the L_o_ domain experiences nanoscopic heterogeneity within microseconds. The effect of heterogeneity is averaged out at longer timescale and thus apparent Brownian motion is observed at longer time interval (>1 ms).

We recorded 166 and 88 diffusion trajectories in the L_d_ and L_o_ domains respectively and calculated their individual time-averaged MSD (Fig. 3). Each trajectory is longer than 1,000 steps. In total, more than 2.3 × 10^6^ and 1.7 × 10^6^ steps were measured in the L_d_ and L_o_ domains respectively, providing a large amount of statistical data for accurate characterization of diffusion. The MSD measured in the L_d_ domain scales linearly with the time interval Δt, indicating the diffusion is Brownian. On the other hand, the MSD measured in the L_o_ domain shows subdiffusion in the microsecond timescale. A crossover from subdiffusion to apparent Brownian motion in the L_o_ domain was observed at ~1 ms with the MSD value of ~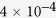 μm^2^, i.e., 20 × 20 nm^2^. It indicates that the molecular diffusion in the L_o_ domain experiences heterogeneity over a length scale <20 nm in the microsecond timescale. At longer timescale of milliseconds, the effect of heterogeneity is averaged out and thus apparent Brownian motion is observed. We stress that experiment without sufficient temporal resolution would not be able to detect subdiffusion in the L_o_ domain but measure apparent Brownian diffusion of slower diffusion rate. We performed the same iSCAT SPT measurement at a lower speed of 1 kHz under otherwise identical conditions, and the diffusion in the L_d_ and L_o_ domains was indeed both Brownian (Fig. S4 and Movie S3). The observed anomalous subdiffusion in microsecond timescale at a length scale <20 nm is truly due to the nanoscopic substructures in the L_o_ domain; it is not systematic artifact (due to GNP labeling or the transient interaction with the supporting substrate) because from the MSD data (Fig. 1e), Brownian diffusion is measured in the benchmark experiment of homogeneous membranes at a length scale as small as 10 × 10 nm^2^ where no confinement is detected. The histograms of the anomalous exponent 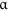 and the diffusion rate 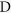 of individual trajectories measured in the L_d_ and L_o_ domains are plotted in Fig. S5, where the diffusion in the L_o_ domain is slow (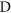 = 0.24 ± 0.12 μm^2^ s^−1^) and subdiffusive (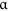 = 0.75 ± 0.16), while diffusion in the L_d_ domains is fast (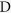 = 1.43 ± 0.50 μm^2^ s^−1^) and much more Brownian (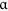 = 0.92 ± 0.08). The measured diffusion rates in the two phases are similar to what were measured previously with dye molecules DiD by fluorescence correlation spectroscopy (FCS)^29^. Nevertheless, we note that the diffusion rate varies between different types of probe molecules, possibly because of their different charges resulting in different strength of electrostatic interaction with the substrate^29^. Anomalous subdiffusion was also observed in the membranes of ternary lipid mixture of DOPC/brain sphingomyelin/cholesterol (40:40:20 mol%) (Fig. S6), suggesting the heterogeneous molecular organization in the L_o_ phase is general, not lipid-specific.

### Transient confinements of saturated lipids in nano-subdomains of the L_o_ phase

**Figure 4 f4:**
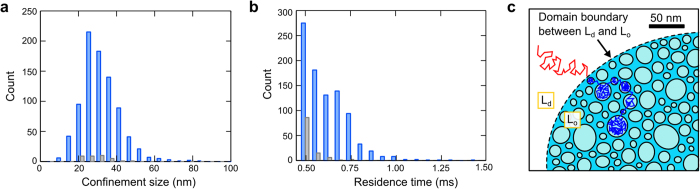
Detection of transient confinements in the L_o_ domain due to nanoscopic substructures. (**a**) Size distributions of the nano-confinements (in diameter) detected from the trajectories measured in the L_o_ domains (in blue) and from the simulated trajectories of Brownian diffusion (in gray). See Methods for the details of detection of nano-confinements. Considerable amount of nano-confinements of 32 ± 10 nm are detected in the L_o_ domains. (**b**) Distributions of the residence time in each confinement. The blue histogram shows the experimental results measured from the L_o_ domains, while the gray histogram shows the control of simulated Brownian diffusion. Single lipid molecules are transiently confined in nano-sized zones inside the raft L_o_ domain for less than 1 ms. (**c**) Schematics of the proposed nanoscopic molecular dynamics and organization in the L_o_ domain. The molecular arrangement in the L_o_ domain is not homogeneous at the nanoscale. There exist numerous subdomains of tens of nanometers, potentially clusters of closely packed saturated lipids of high melting temperature. These subdomains strongly influence diffusion of molecules. For illustration purpose, an artificial diffusion trajectory is plotted showing fast and free diffusion in the L_d_ phase (red) and transient confinements in the nano-subdomains of the L_o_ phase (blue).

To gain more insights of the observed subdiffusion, we looked carefully into the diffusion trajectories in the L_o_ domains. As shown in Fig. 2e, there are numerous clusters of localizations in the trajectories measured in the L_o_ domain. Further analysis shows that many of these clusters are transient confinements of the lipid in nano-sized zones (see Methods)^31^. We analyzed the size of confinements and the residence time in each confinement from all trajectories measured in the L_o_ domains (see Methods, Fig. 4a[Fig f4]b). We set relatively high criteria (99% confidence) for detecting transient confinement so that false detection of stochastic confinement-like Brownian motion is mostly avoided (Fig. 4a[Fig f4]b). Our analysis shows that there are considerable amount of nano-confinements in the L_o_ phase. The average residence time is 0.62 ms, and the average size of the confinement is 32 nm in diameter. We note that there could be much more confinements that are very transient and of very small sizes, and their reliable detection requires measurements of even higher spatiotemporal resolution. In fact, our MSD data of the L_o_ domains (Fig. 3) show anomalous diffusion spanning the length scale from (6 nm)^2^ to (20 nm)^2^, implying the membrane organization of the L_o_ domain is heterogeneous over the nanoscale. As the probe lipid being tracked in our experiment is the saturated lipid biotin-cap-DPPE, these nano-subdomains which transiently trap the probe lipid are likely to be clusters of closely packed saturated lipids DPPC of similar molecular structures and properties. Based on our experimental observation, we propose a potential model of nanoscopic molecular arrangement in the L_o_ domain (Fig. 4c): inside the L_o_ domain, there exist numerous subdomains smaller than tens of nanometers that strongly influence the molecular diffusion. This model has been suggested by the nuclear magnetic resonance (NMR) spectroscopy studies showing that the formation of the L_o_ phase is mainly governed by the interaction between saturated lipids of high melting temperature (e.g., sphingolipids) and therefore nano-sized clusters may exist in the L_o_ domain^32^. Molecular dynamic simulation has also predicted anomalous diffusion and substructures of saturated lipids in the L_o_ phase at nanosecond timescale^33^,^34^.

## Discussion

The fact that the molecular organization in the L_o_ phase is heterogeneous at the nanoscale is striking. It has been known that the cholesterol in the L_o_ phase disturbs the packing of lipids that are otherwise in gel phase^14^. The phase diagram of lipid mixture is changed after adding cholesterol, revealed by NMR spectroscopy and differential scanning calorimetry (DSC) measurements^35^,^36^. The intermediate phase state induced by the cholesterol, e.g., the L_o_ phase, is speculated to play critical role in biological membranes via reducing the tendency of the lipids to form separate L_d_ and gel phases^37^ and at the same time stabilizing putative functional nanodomains in cell membranes (rafts)^38^. However, there has been no experimental data showing how cholesterol disturbs the packing of lipids at the nanoscale and at the same time sustains the integrity of the L_o_ domain. In this work, we show explicitly that there are numerous nano-subdomains in the L_o_ phase. Although we do not have further evidence to confirm exactly what those nano-subdomains are consisted of at the present stage, based on our observation of nano-confinements in the trajectories, they are likely to be densely packed saturated lipids DPPC (the lipid that has higher melting temperature in the ternary mixture) which have similar molecular structures as our probe lipids DPPE. Cholesterol and a low density of DiPhyPC (the lipid of lower melting temperature) fill the gaps between the nano-subdomains. Molecular dynamic simulation has also indicated the existence of nano-clusters of saturated lipids in the L_o_ domains^34^.

We notice that there have been several measurements of the topography of the L_d_ and L_o_ coexisting supported bilayer membranes by using atomic force microscopy (AFM)^29^,^39^,^40^. However, in those AFM studies, no nano-substructures were observed in the L_o_ phase. We think it may be because of the following reasons. First, detection of the nano-substructures of the L_o_ phase requires high-resolution AFM measurement. We estimate the height difference between the nano-substructures and the surrounding area to be <0.8 nm, considering the height difference between L_d_ and L_o_ phases is ~0.8 nm^29^,^39^,^40^. Regarding the lateral size, according to our MSD data and transient confinement analysis, the size of the substructures is smaller than tens of nanometers. As mentioned in the text, we believe that there are smaller subdomains that are not detected by the technique presented in this work. In order to explicitly map the dense and fine substructures of the L_o_ phase, resolution of subnanometer may be required. Second, the nano-substructures may be dynamic which requires high-speed AFM to resolve them. Third, the nano-substructures may be fragile and therefore perturbation introduced by the AFM tip during measurement should be minimized. As the sensitivity and spatiotemporal resolution of AFM technique has been improved rapidly in recent years, high-resolution and high-speed AFM with subnanometer resolution^41^ and other near field techniques show promise of providing complementary evidence of the molecular organization of the L_o_ phase.

It is worth noting that a very similar study was conducted aiming to resolve the nanoscopic molecular organization of the L_o_ phase by using a superresolution optical technique of stimulated emission depletion – fluorescence correlation spectroscopy (STED-FCS)^42^. That measurement did not detect anomalous diffusion in the L_o_ phase and concluded that the slow diffusion of the L_o_ phase is mainly due to the tight molecular packing rather than causing anomalous subdiffusion over a length scale >40 nm. The high-speed iSCAT SPT data presented in this work further extend the detection resolution down to a few nanometers and show that at the smaller length scale (<30 nm) there are nano-substructures of the L_o_ phase that strongly influence lipid diffusion, causing subdiffusion via transient confinements. High spatiotemporal resolution is ultimately necessary in order to resolve nanoscopic structures in dynamic membrane systems.

While our measurements were performed in the L_o_ domains of model membranes, the measured inherent molecular interaction and the resulting molecular organization at the nanoscale are expected to be closely related to that of lipid rafts in cell membranes. It has been shown that biological membranes are heterogeneous at the nanoscale^43^,^44^, and the size of lipid rafts could be smaller than tens of nanometers^5^,^6^. Considering the nano-sized subdomains of the L_o_ phase observed in our experiments, molecular organization of rafts in cell membranes could be highly heterogeneous. Also, we reveal that molecular dynamics in the L_o_ phase is strongly influenced by the nano-subdomains, and therefore these nano-substructures may readily be responsible for determining the partition of molecules in lipid rafts. The raft-associated proteins could be trapped between subdomains of densely packed saturated lipids and surrounded by high density of cholesterol. The nanoscopic membrane environment created by the nano-subdomain of rafts is thus unique, which illustrates the essentials of lipid rafts of L_o_ phase whose roles cannot be fulfilled solely by the gel-phase or L_d_-phase domains.

In summary, by using ultrahigh-speed iSCAT optical technique together with small nanoparticles as labels, we have investigated single molecule diffusion in bilayer model membranes with nanometer spatial precision and microsecond temporal resolution. Diffusion of single nanoparticles on supported bilayer membranes has been studied by high-speed iSCAT microscopy before^22^,^23^. In the previous work, anomalous diffusion, multimobility and also nano-confinements of the particles were observed due to the multiple lipid binding sites on the particle, clustering of the lipids, and the substrate effects, which complicate the investigation of single-molecule dynamics in the membrane. Addressing these issues, in this work, we promote the probability of monovalent binding by saturating the multiple binding sites on the particle. In addition, we prepared bilayer membranes on atomically flat mica substrate, which significantly reduces the heterogeneous substrate effect. We also used a well-behaved probe lipid, biotin-cap-DPPE, that does not form clusters and diffuses freely in homogeneous membrane without transient confinements. Motion of the single particles faithfully represents the diffusion of single molecules with nanometer precision as supported by the data of Brownian diffusion observed in the homogeneous membrane down to microsecond timescale. We have further employed iSCAT SPT to investigate molecular dynamics and nanoscopic organization of raft-mimetic L_o_ domains. We have found transient confinements of lipid molecules due to the inherent nano-substructures of the L_o_ domains that have not been resolved before, providing a new view of molecular organization in the L_o_ phase at the nanoscale. These nano-subdomains of the L_o_ phase are likely to be responsible for governing the selection, partition and confinement of raft-associated molecules in raft domains of cell membranes.

## Methods

### iSCAT microscope

In iSCAT microscope, illumination in wide field was made by focusing a 532 nm laser beam (Finesse Pure, Laser Quantum) at the back focal plane of a high NA microscope objective (UPLSAPO 100X, NA 1.4 Olympus). Part of the excitation (~0.6%) was reflected at the mica-water interface due to the refractive index mismatch, serving as a coherent reference beam for iSCAT imaging. The transmitted light illuminated the GNP. The backscattered signal from the GNP together with the reflective reference beam was collected by the same objective and imaged onto a high-speed CMOS camera (Phantom v711, Vision Research). The overall optical magnification of iSCAT imaging was 416.7, corresponding to pixel resolution of 48 × 48 nm^2^ per pixel. Destructive interference between the scattering signal of GNP and the reference beam results in a dark spot in the iSCAT image. We recorded iSCAT videos at 50 kHz for a few seconds during which GNPs diffuse on the membrane. Excitation intensity was ~10 kW cm^−2^, causing a minor temperature increase on the surface of GNP by ~0.3 K. The iSCAT microscope was equipped with epi-fluorescence imaging in parallel. Fluorescence images were taken by a sensitive camera (EMCCD; iXon Ultra 897, Andor) at 1 Hz simultaneously with iSCAT imaging. The overall optical magnification of fluorescence imaging was 166.7, corresponding to pixel resolution of 96 × 96 nm^2^ per pixel. The iSCAT and fluorescence images were aligned by localizing immobile 100 nm fluorescent particles on coverglass simultaneously in both channels before the membrane experiments. The relative sub-pixel displacement between the iSCAT and fluorescence images is corrected in the final overlay of the fluorescence image of the membrane domains and the iSCAT trajectories. The precision of the overlay is ~4 nm, limited by the localization precision of the fluorescent particles.

### Preparation of reconstituted supported bilayer membranes on mica and GNP labeling

We used mainly two types of lipid bilayer membranes in this work. The raft-containing membrane consists of 40:40:20 mol% of 1,2-diphytanoyl-sn-glycero-3-phosphocholine (DiPhyPC), 1,2-dipalmitoyl-sn-glycero-3-phosphocholine (DPPC), and cholesterol (Chol). The homogeneous membrane was made by DiPhyPC. Both types of membranes composed of 1 mol% biotinylated probe lipid analogues (1,2-dipalmitoyl-sn-glycero-3-phosphoethanolamine-N-(cap biotinyl); biotin-cap-DPPE) and 0.1 mol% fluorescent carbocyanine dyes. Since both phospholipids (DiPhyPC and DPPC) are saturated, the membranes have less chance to be photo-oxidized during measurement. These lipid molecules and cholesterol were purchased from Avanti (Avanti Polar Lipids Inc., Alabaster, Alabama, USA.) The orange—red fluorescent dye, 1,1′-dioctadecyl-3,3,3′,3′-tetramethylindocarbocyanine perchlorate (DiI-C18), was purchased from Life Science. Note that DiI-C18 is a lipophilic membrane stain that prefers to partition in the L_d_ phase of membranes. Therefore, the dark regions of the fluorescence images represented L_o_ domains.

The supported lipid bilayers (SLBs) were formed by vesicles fusion method^45^. All lipids were dissolved in chloroform, and they were dried to a film by nitrogen and kept under vacuum for at least 1 hour right before use. The dried lipid film was hydrated to a lipid concentration of 1 mg mL^−1^ in bilayer buffer solution (10 mM HEPES, 200 mM NaCl_2_, and 2 mM CaCl_2_, PH 7.0) for 1 hour at 60 °C. Multilamellar vesicles (MLVs) were then formed by vortexing the lipid solution. This cloudy solution was diluted by buffer solution to 0.25 mg mL^−1^ and further sonicated for 10 minutes at 30% power in a tip sonicator at 60 °C (Q700, Qsonica LLC., USA). Through this process, the MLVs were broken into small unilamellar vesicles (SUVs). Next, the lipid solution was centrifuged for 20 minutes at 16,000 g, and the SUVs in the supernatant were carefully extracted without the debris and the remaining MLVs at the bottom. The clear solution of SUVs were stored at 4 °C and used within 48 hours.

To form SLBs, a mica disc (Ted Pella Inc, USA) was first glued on a cleaned coverslip with the optical adhesive (Norland Products Inc, USA). Then, the SUV solution was introduced to a freshly cleaved thin mica disc (a few microns thick) in a dish-type chamber (Live Cell Instrument, Seoul, Korea) for at least 90 minutes at 60 °C. The SLBs were gently washed with buffer solution to remove excess SUVs. Note that all the procedures were done at room temperature while preparing homogeneous membranes. Finally, 20 nm GNPs functionalized with streptavidin (BBI Solutions, Cardiff, U.K.) were added to the SLB for labeling the biotinylated probe lipid analogue at the hydrophilic headgroup. The streptavidin as the crosslinker is a tetramer that is able to bind to more than one biotin-cap-lipid in the membrane. In order to make monovalent labeling more probable, we estimated the number of available streptavidin binding sites on the GNP and then saturated them by mixing an excess amount of biotin molecules into the GNP colloidal solution before introducing them to membrane labeling. Stochastically, there should be one or no available binding site for most GNPs.

### Single-particle tracking (SPT)

In order to localize the GNP with nanometer spatial precision, it is necessary to start with a clean iSCAT image of the GNP with a highly uniform background. Therefore, the inhomogeneous pixel response of the CMOS camera, the inevitably non-uniform light illumination, and the weak iSCAT signal of the membrane domains need to be removed. These effects are relatively stationary within our observation timescale, especially compared with the fast diffusing GNP on a membrane. We extracted a stationary background from a stack of images by calculating the temporal median value of each pixel of the image^22^. Background-free iSCAT images were obtained after correcting the extracted stationary background from the raw images. After background removal, a diffraction-limited point spread function as a dark spot of a GNP can be clearly visualized. The center of the dark spot in every frame was determined by fitting the measured point spread function with a two-dimensional Gaussian function with a home-written code in MATLAB. Diffusion trajectories were obtained by connecting the corresponding localizations in the consecutive frames. Typical localization precision of single 20 nm GNP diffusing on the membrane recorded at 50 kHz is 3 nm (see Fig. S2).

### Calculation of time averaged MSD for characterizing diffusion behaviors

For every trajectory, the two-dimensional MSD as a function of time interval Δt ranging from 20 μs to 20 ms was calculated according to


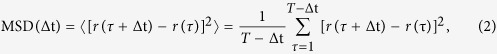


where 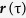 is the position of the particle at time 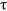 and 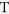 is the length of the trajectory. The diffusion rate, 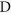, is calculated from the first two data points of the MSD^46^, which refers to the microscopic diffusion rate measured at 20 μs time interval.

To analyze whether diffusion deviates from Brownian motion, the MSD data were fitted by a model of anomalous diffusion as shown in Eq. (1) where 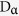, the anomalous transport coefficient, 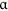, the anomalous exponent, and 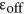, an offset are the three fitting parameters. Notice that the offset 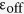 in the MSD is the result of dynamic localization uncertainty, determined by the localization precision of the particle, the diffusion rate, and the exposure time^28^. Error in estimating the value of 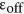 can cause misinterpretation of the data for anomalous diffusion, which is especially critical in high-speed measurements where the true displacements can be comparable to the dynamic localization uncertainty. We found that the most reliable way of finding the value of 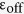 is to fit it as a free parameter for individual MSD. After subtracting the offset from the MSD, the double logarithmic plot of the corrected MSD versus time interval has a slope of 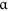. Based on the value of 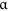, the diffusion characteristics of the particle can be classified: 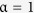 for Brownian diffusion, 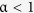 and 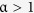 indicating sub- and superdiffusion, respectively.

Furthermore, to statistically elaborate the dynamic motion of multiple particles we ensemble averaged the MSD data by weighting the data according to the length of each trajectory,


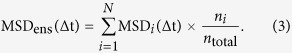


Here, MSD_*i*_(Δt) is the MSD for the 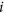
th trajectory with 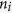 steps and 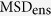
(Δt) is the ensemble averaged MSD of *N* trajectories with 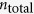 steps in total.

To estimate a transient diffusion constant, we chopped the original trajectory into segments of 146 steps and computed the diffusion rate from the MSD of each segment.

### Detection of transient confinements

Transient confinements occurring in the L_o_ domains were detected by the algorithm introduced by Simson *et al*.^31^. Briefly, we calculate the probability level *L* of a lipid of diffusion rate 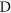 staying within a region of radius 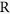 within a time window 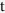 as


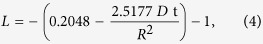


where 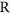 is defined by the point in the time window with the largest displacement from the starting point. In our analysis, we chose the time window to be 25 steps. Transient confinements were defined as the probability level 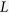 surpassed a critical probability level 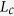. In our work, the value of 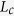 was chosen to be one, corresponding to 99% confidence that the detected transient confinement is indeed restricted non-Brownian motion. The residence time in a confinement is defined as the duration when 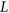 is successively larger than 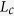. In order to minimize the disturbance of inevitable localization errors in the detection, we calculate the moving average of the raw probability level as a function of time with an averaging window of 10 steps. Following the procedures described above, we reliably detected a large number of events of transient confinement in the L_o_ domain. To further affirm the detected confinements in the L_o_ domains are not rare stochastic behavior of Brownian motion, we performed the identical detection with the computer-generated Brownian diffusion of the same trajectory length, diffusion rate 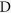, and localization errors. We confirmed that the detected transient confinements from simulated Brownian motion is negligible, indicating that the confinements measured in the L_o_ domains were real.

## Additional Information

**How to cite this article**: Wu, H.-M. *et al*. Nanoscopic substructures of raft-mimetic liquid-ordered membrane domains revealed by high-speed single-particle tracking. *Sci. Rep.* **6**, 20542; doi: 10.1038/srep20542 (2016).

## Supplementary Material

Supplementary Information

Supplementary Movie S1

Supplementary Movie S2

Supplementary Movie S3
