# Supplementary material for: Nanoscopic substructures of raft-mimetic liquid-ordered membrane domains revealed by high-speed single-particle tracking
Source: Sci Rep. 2016 Feb 10;6:20542. doi: 10.1038/srep20542 (PMC4997016; doi:10.1038/srep20542)
Supplement: Supplementary Information [file srep20542-s1.pdf]

Supplementary Information

Nanoscopic substructures of raft-mimetic liquid-ordered membrane domains revealed by high-speed single-particle tracking

*Hsiao-Mei Wu, Ying-Hsiu Lin, Tzu-Chi Yen, Chia-Lung Hsieh**

Supplementary materials

A. iSCAT optical microscope setup and alignment
B. Localization precision of single 20 nm GNP at 50,000 fps
C. Verification of loading effect of 20 nm GNPs on single lipid molecules in the membrane
D. Brownian diffusion of single lipid molecules (biotin-cap-DPPE) in the L_d and L_o domains of DiPhyPC/DPPC/cholesterol (40:40:20 mol%) observed at 1,000 fps
E. Statistical diffusion characteristics of single lipids (biotin-cap-DPPE) in the L_d and L_o domains of DiPhyPC/DPPC/cholesterol (40:40:20 mol%) observed at 50,000 fps
F. Diffusion of single lipids (biotin-cap-DPPE) in raft-containing membranes of DOPC/brain sphingomyelin/cholesterol (40:40:20 mol%) observed at 50,000 fps

Supplementary movies

Movie S1: Free diffusion of single 20 nm GNPs labeled to single probe lipid molecules (biotin-cap-DPPE) in a homogeneous DiPhyPC membrane.

Movie S2: Reconstructed diffusion trajectory of single probe lipid molecule repeatedly crossing the domain boundary between L_d and L_o domains, recorded at 50 kHz. The trajectory is plotted in red and blue when the lipid is in L_d and L_o domains respectively.

Movie S3: Diffusion of single 20 nm GNP labeled to single lipid molecule in a raft-containing membrane, recorded at 1 kHz. The trajectory is plotted in red and blue when the lipid is in L_d and L_o domains respectively. Domain boundaries are drawn with yellow dashed lines.

A. iSCAT optical microscope setup and alignment

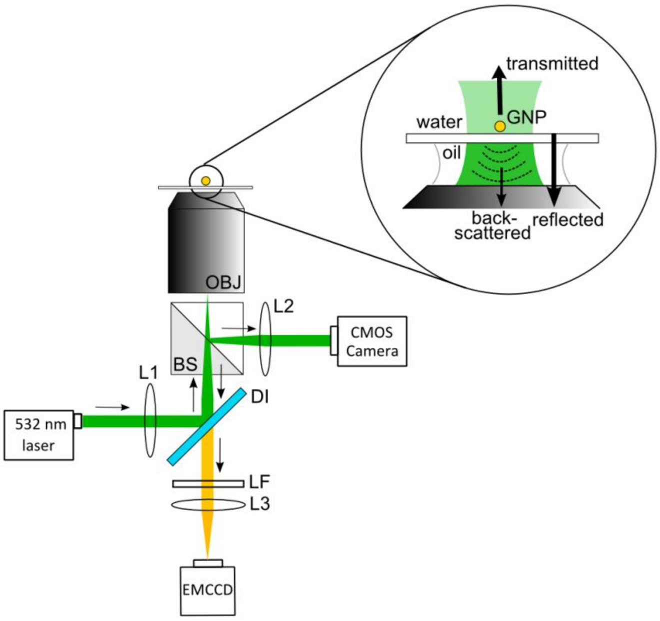

Fig. S1. Schematics of the iSCAT optical microscope apparatus. L1, L2, L3 are achromatic doublet lens of focal lengths of 20 cm, 75 cm, and 30cm respectively. BS is a 50:50 non-polarizing beamsplitter; DI is a long-pass dichroic mirror that has a cutoff wavelength of 560 nm (Semrock, cat. No. FF560-Di01); LF is a long-pass edge filter (Semrock, cat. No. BLP01-532R-25).

L1 focuses the laser beam (~2 mm beam diameter) at the back focal plane of the microscope objective (UPLSAPO 100X, NA 1.4 Olympus), and thus creates a wide-field illumination of an area of ~10 μm in diameter on the sample. L2 and L3 project the iSCAT image and the fluorescence image of the same area of the sample onto the high-speed CMOS camera (Phantom v711) and the EMCCD camera (Andor iXon Ultra 897) respectively. These two cameras are aligned by imaging the same 100 nm fluorescent beads via scattering and fluorescence simultaneously on the sample. For iSCAT imaging, the microscope objective and lenses are carefully aligned (normal beam incidence) such that optical aberration and astigmatism are minimized. The orientation of the beamsplitter is however intentionally tilted away from the perfectly normal incidence, such that the undesirable reflection from the surface of the beamsplitter is spatially separated from the iSCAT signal. On the high-speed CMOS camera, the dominant signal is the reflection at the interface between the supporting substrate and the water.

B. Localization precision of single 20 nm GNP at 50,000 fps

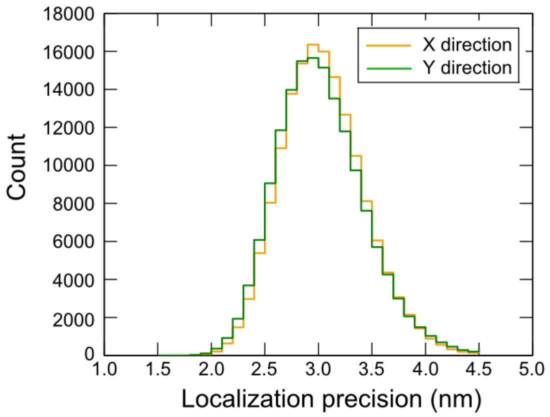

Fig. S2. Histogram of localization precision in two lateral directions of single 20 nm GNP labeled to lipid molecules diffusing in the membrane with a mobility of ~1.5 $\mu m^2/s$ measured at 50k fps, showing identical distributions centered at 3 nm. Here, the localization precision denotes the quality of fitting of the experimental iSCAT image with a two-dimensional (2D) Gaussian profile[1]. It is worth noticing that localization precision of a diffusing GNP is fundamentally different from the precision of an immobile GNP because diffusion blurs the image even within short exposure time of 20 µs[2]. As a result, we estimate our localization precision directly from the quality of iSCAT diffusion images via 2D Gaussian fitting. The localization precision of 3 nm also matches with the an alternative estimation from the offset of the MSD results[3], indicating the value is correct.

C. Verification of loading effect of 20 nm GNPs on single lipid molecules in the membrane

As the 20 nm GNP is much larger than the lipid molecule (~1 nm), it is critical to ensure the GNP does not impede the motion of the lipid being labeled. To verify the possible loading effect of the GNP, we compare the diffusion of single lipids tagged by dye-labeled streptavidin and GNP-streptavidin conjugate (see Fig. S3 below). The bilayer membrane consisting of 1 mol% of biotin-cap-DOPE and 99 mol% of DOPC of L_d phase was prepared on a glass substrate. Diffusion trajectories of single dye-labeled streptavidins (atto532-streptavidin) tagged to lipid molecules were measured in fluorescence at 100 fps. Diffusion of single 20 nm GNP-streptavidin conjugates was measured by iSCAT SPT at 1,000 fps. Diffusion rate was found from the time-averaged MSD calculated from individual trajectories. From the histograms, the dye-labeled streptavidin and GNP streptavidin conjugate show the same distributions of diffusion rates, indicating that the load of 20 nm GNP does not influence the diffusion of lipid molecules.

It should be noticed that although the GNP is much larger than the lipid molecule, the GNP is surrounded by water and its diffusion rate in water is ~ 20 $\mu m^2/s$ that is much greater than the diffusion rate of lipids in the membrane (~ 1 $\mu m^2/s$). This is because water is about 100 times less viscous than the membrane. Therefore, hydrodynamic loading of a 20 nm GNP to a lipid molecule does not slow down the overall diffusion. Furthermore, the loading effect should be independent of the material composition of the particle because the diffusion rate of the particle is determined by its size not by its density. While 20 nm GNP shows no labeling effects, it has been reported that using large particles (>40 nm) as labels can affect the diffusion of lipid molecules[4]. Besides, close attention needs to be paid to the monovalent labeling of the particle to the lipid molecule for investigation of single-lipid diffusion[5]. In our experiments, we promoted

the monovalent labeling by saturating the available binding sites on the particle with excess amount of biotin molecules in solution prior lipid labeling (see Methods).

In our precise measurement with nanometer precision, it becomes important to consider if the position of the GNP provided by iSCAT SPT represents the position of the labeled lipid molecule. The GNP labeled to the lipid is expected to have a small degree of freedom to rock on the membrane due to the flexibility of the cross linker (streptavidin and biotin). Even when capturing the iSCAT image of the GNP at high speed, the GNP still rock restlessly in water within each exposure (of microseconds), and therefore the iSCAT image is actually a blurred image of the rocking GNP centered at the labeled lipid molecule. Indeed, the lipid molecule also constantly moves due to diffusion in each exposure but at a much slower rate. As a result, because of the large mismatch between the diffusion rates of the GNP in water and the lipid in the membrane, the position of the GNP determined from the iSCAT image is still able to faithfully represent the position of the labeled lipid molecule. This argument is supported by the Gaussian step size distribution (Fig. 1d) and the observed Brownian motion of the GNP labeled to the lipid diffusing in homogeneous membrane (Fig. 1e).

In addition to the mass loading of the GNP, one should also consider the possible influence of the crosslinker, in our case, streptavidin. Due to the geometry of streptavidin, one streptavidin could potentially bind to two probe lipids in the plane of the membrane. As described in Methods, we tried to avoid such bivalent binding by saturating the available binding sites with an excess amount of free biotin molecules in the solution. Stochastically, there should be one or no available binding site per particle for most GNPs. We found that the diffusion rate of GNP-streptavidin conjugates labeled on the membrane supported on coverglass is indeed increased after this saturation step, indicating that the saturation treatment is effective. However, we did not see much effect on the membrane supported on mica: both saturated and unsaturated GNPs diffuse fast on the membrane supported on mica. We believe this is because the mica substrate is defect-free and atomically flat, and therefore the Saffman–Delbrück model holds[6]. That is, even if the GNP labels two lipids, the diffusion rate should be almost identical (because the difference in membrane inclusion of one and two lipids is very small). We also want to point out that diffusion and domain partition of single lipids are very sensitive to conjugation and labeling[7]. In the present work, we have verified that streptavidin labeling to biotin-cap-lipids does not cause anomalous diffusion and thus suitable for investigating nanoscopic membrane organization. Whether streptavidin labeling modifies the absolute lipid diffusion rate or domain partition remains to be investigated.

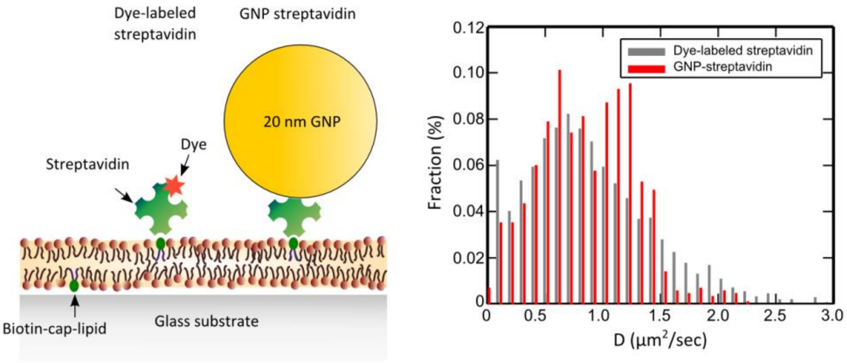

Fig. S3. Comparison of single lipid diffusion tagged by dye-labeled streptavidin and GNP-streptavidin conjugate.

D. Brownian diffusion of single lipid molecules (biotin-cap-DPPE) in the L_d and L_o domains of DiPhyPC/DPPC/cholesterol (40:40:20 mol%) observed at 1,000 fps

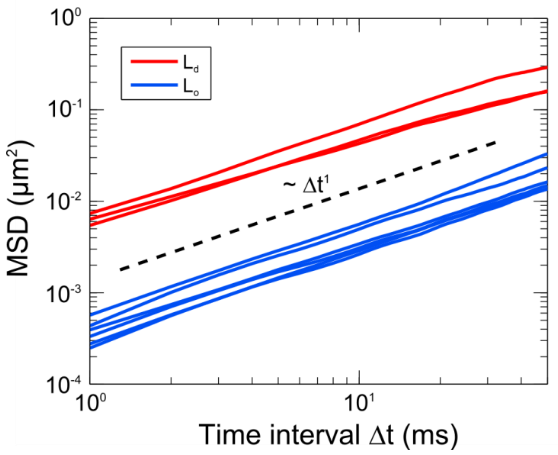

Fig. S4. Time-averaged MSD of single lipid diffusion in the L_d domains (red) and L_o domains (blue) measured at 1,000 fps, both showing free diffusion (~ Δt^1) from 1 ms to 50 ms timescale. This result confirms that the heterogeneous interaction of nano-structures on lipid diffusion is averaged out over time, and the lipid exhibits apparent Brownian diffusion at longer timescale in milliseconds. High temporal resolution (in microseconds) is necessary to detect anomalous diffusion caused by transient interaction with nanoscopic substructures in the raft L_o domain (as shown in Fig. 3).

E. Statistical diffusion characteristics of single lipids (biotin-cap-DPPE) in the L_d and L_o domains of DiPhyPC/DPPC/cholesterol (40:40:20 mol%) observed at 50,000 fps

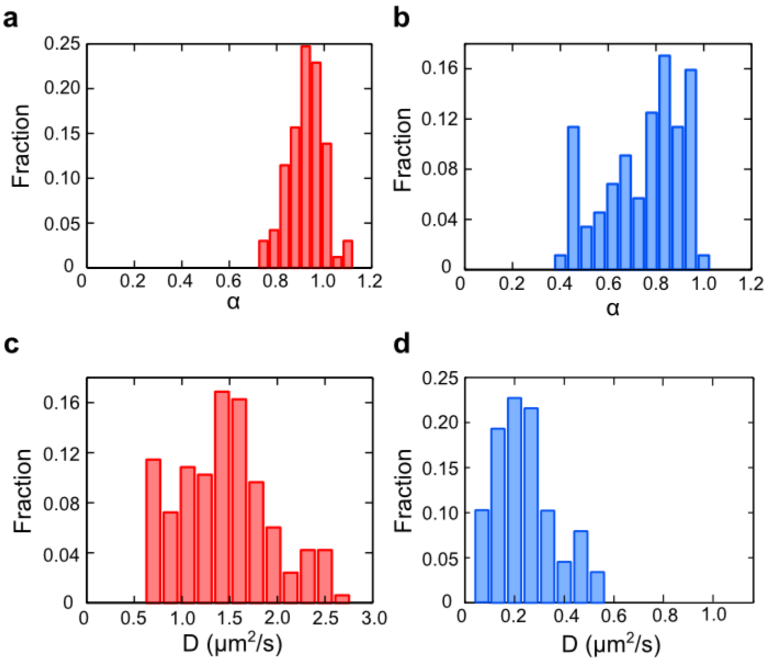

Fig. S5. (a)(b) Histograms of the measured anomalous exponent α in the non-raft L_d domains (a) and raft L_o domains (b). Diffusion in the L_d domain has a value of α close to one ($\alpha = 0.92 \pm 0.08$), indicating the diffusion is nearly Brownian. On the other hand, diffusion in the L_o domain has a smaller value of α ($\alpha = 0.75 \pm 0.16$), indicating the diffusion is subdiffusive. (c)(d) Histograms of the measured diffusion rate D in the non-raft L_d domains (c) and raft L_o domains (d). Diffusion rate of single lipid molecule in the L_d domain ($D = 1.43 \pm 0.50\ \mu m^2/s$) is ~ 6 times greater than in the L_o domain ($D = 0.24 \pm 0.12\ \mu m^2/s$).

F. Diffusion of single lipids (biotin-cap-DPPE) in raft-containing membranes of DOPC/brain sphingomyelin/cholesterol (40:40:20 mol%) observed at 50,000 fps

Lipid membrane of DOPC/brain sphingomyelin/cholesterol (40:40:20 mol%) is commonly used to study L_d and L_o phase domains where micron-sized domains can readily form for fluorescence observation. However, both the DOPC and sphingolipids are prone to photooxidation at lipid double bonds, and it has been reported that large raft formation can be promoted when photooxidation proceeds[6]. It is not clear how the lipid peroxides influence the molecular organization in the L_d and L_o domains. Therefore, in this work we mainly used membranes of DiPhyPC/DPPC/cholesterol for the investigation of diffusion. Nevertheless, we also tried high-speed iSCAT SPT in the DOPC/brain sphingomyelin/cholesterol in the deoxygenated solutions (10 mM HEPES, 200 mM $NaCl_2$, 2 mM $CaCl_2$, and 100 mM ascorbic acid). We again observed anomalous subdiffusion in the raft L_o domain and Brownian diffusion in the non-raft L_d domain (Fig. S6).

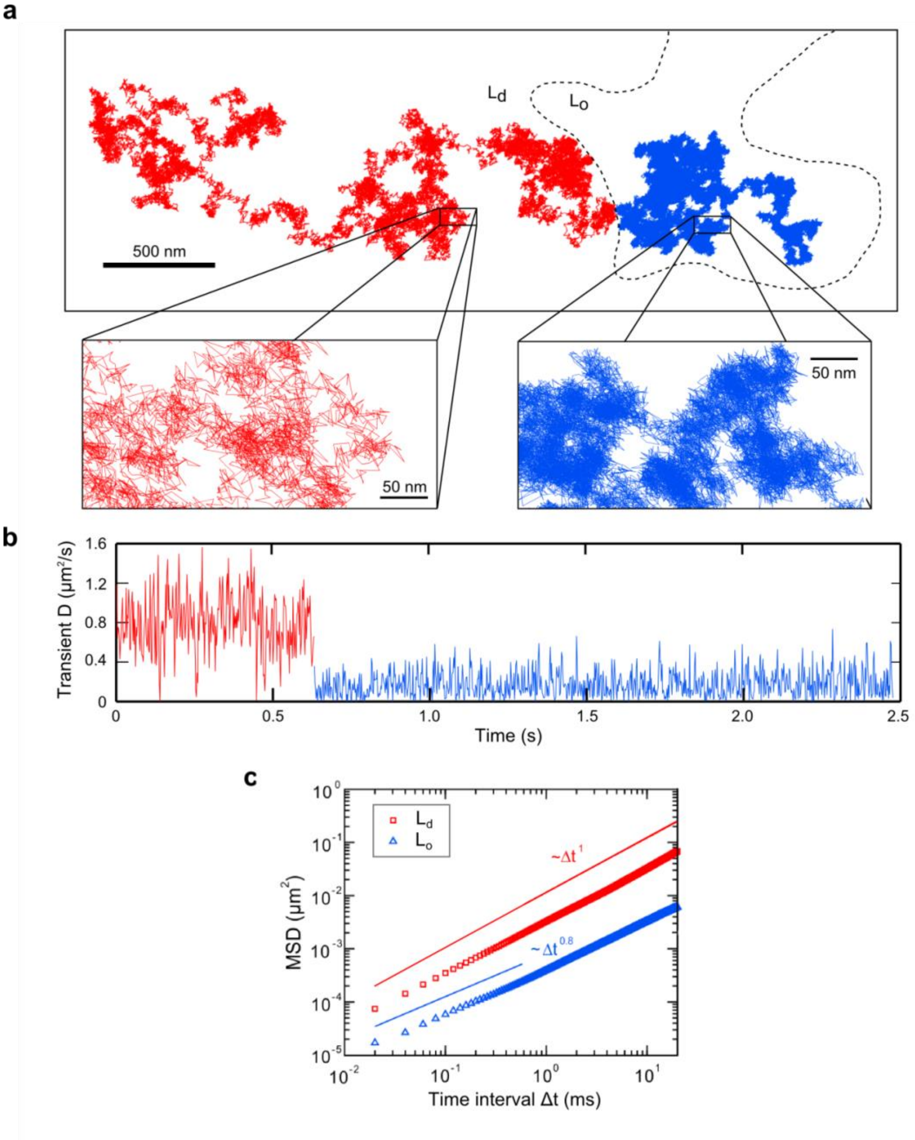

Fig. S6. Diffusion of single lipids (biotin-cap-DPPE) in raft-containing membranes of DOPC/brain sphingomyelin/cholesterol (40:40:20 mol%) observed at 50k fps. (a) Continuous diffusion trajectory of a lipid starting in the L_d domain and entering the L_o domain. Insets are close-up views of the trajectories, showing distinct diffusion behaviors in the L_d and L_o domains. (b) Transient diffusion rate decreases abruptly when the lipid enters the L_o domain. (c) Time-averaged MSD of the diffusion in the L_d and L_o domains respectively. Diffusion is Brownian in the L_d domain, and it becomes subdiffusive in the L_o domain in the microsecond timescale.

References

1 Bobroff, N. Position measurement with a resolution and noise-limited instrument. *Rev. Sci. Instrum.* **57**, 1152-1157, (1986).

2 Ritchie, K. *et al.* Detection of non-Brownian diffusion in the cell membrane in single molecule tracking. *Biophys. J.* **88**, 2266-2277, (2005).

3 Martin, D. S., Forstner, M. B. & Käs, J. A. Apparent subdiffusion inherent to single particle tracking. *Biophys. J.* **83**, 2109-2117, (2002).

4 Mascalchi, P., Haanappel, E., Carayon, K., Mazères, S. & Salomé, L. Probing the influence of the particle in Single Particle Tracking measurements of lipid diffusion. *Soft Matter* **8**, 4462-4470, (2012).

5 Lee, G. M., Ishihara, A. & Jacobson, K. A. Direct observation of brownian motion of lipids in a membrane. *Proceedings of the National Academy of Sciences* **88**, 6274-6278, (1991).

6 Saffman, P. G. & Delbrück, M. Brownian motion in biological membranes. *Proceedings of the National Academy of Sciences* **72**, 3111-3113, (1975).

7 Sezgin, E. *et al.* Partitioning, diffusion, and ligand binding of raft lipid analogs in model and cellular plasma membranes. *Biochimica et Biophysica Acta (BBA) - Biomembranes* **1818**, 1777-1784, (2012).

8 Ayuyan, A. G. & Cohen, F. S. Lipid peroxides promote large rafts: effects of excitation of probes in fluorescence microscopy and electrochemical reactions during vesicle formation. *Biophys. J.* **91**, 2172-2183, (2006).
